# Working With the Encounter: A Descriptive Account and Case Analysis of School-Based Collaborative Mental Health Care for Refugee Children in Leuven, Belgium

**DOI:** 10.3389/fpsyg.2022.806473

**Published:** 2022-03-07

**Authors:** Caroline Spaas, Siel Verbiest, Sofie de Smet, Ruth Kevers, Lies Missotten, Lucia De Haene

**Affiliations:** ^1^Parenting and Special Education Research Unit, Faculty of Psychology and Educational Sciences, KU Leuven, Leuven, Belgium; ^2^Studies in Performing Arts and Media, Faculty of Arts and Philosophy, University of Ghent, Ghent, Belgium

**Keywords:** refugee children and families, development, mental health, collaborative mental health care, school-based mental health care, school–family relations, transcultural assessment, intervention

## Abstract

Scholars increasingly point toward schools as meaningful contexts in which to provide psychosocial care for refugee children. Collaborative mental health care in school forms a particular practice of school-based mental health care provision. Developed in Canada and inspired by systemic intervention approaches, collaborative mental health care in schools involves the formation of an interdisciplinary care network, in which mental health care providers and school partners collaborate with each other and the refugee family in a joint assessment of child development and mental health, as well as joint intervention planning and provision. It aims to move away from an individual perspective on refugee children’s development, toward an engagement with refugee families’ perspectives on their migration histories, cultural background and social condition in shaping assessment and intervention, as such fostering refugee empowerment, equality, and participation in the host society. Relating to the first stage of van Yperen’s four-stage model for establishing evidence-based youth care, this article aims to engage in an initial exploration of the effectiveness of a developing school-based collaborative mental health care practice in Leuven, Belgium. First, we propose a detailed description, co-developed through reflection on case documents, written process reflections, intervision, an initial identification of intervention themes, and articulating interconnections with scholarly literature on transcultural and systemic refugee trauma care. Second, we engage in an in-depth exploration of processes and working mechanisms, obtained through co-constructed clinical case analysis of case work collected through our practice in schools in Leuven, Belgium. Our descriptive analysis indicates the role of central processes that may operate as working mechanisms in school-based collaborative mental health care and points to how collaborative mental health care may mobilize the school and the family-school interaction as a vehicle of restoring safety and stability in the aftermath of cumulative traumatization. Our analysis furthermore forms an important starting point for reflections on future research opportunities, and central clinical dynamics touching upon power disparities and low-threshold access to mental health care for refugee families.

## Introduction

Scholars in the field of refugee mental health care increasingly point toward the value of community-based psychosocial interventions (e.g., Rousseau and Measham, 2007; Nadeau et al., 2017). For refugee children, the school is viewed as a meaningful locus of both preventive and curative psychosocial care provision (e.g., Tyrer and Fazel, 2014; Sullivan and Simonson, 2016; Reynolds and Bacon, 2018). First, scholars argue how school-based mental health care may ensure low-threshold access to psychosocial care and counteract significant barriers in refugee communities’ participation in regular psychosocial services. While refugee children are consistently documented as being an at-risk group for the development of mental health difficulties (e.g., Hodes and Vostanis, 2019), refugee communities display a strongly decreased participation in mental health provisions, with social isolation, cultural stigma, as well as institutional dynamics in mental health care services explaining this general underutilization of regular mental health services (e.g., Colucci et al., 2015; Place et al., 2021). Here, studies show how schools might constitute a safe place for mental health care provision for refugee children, and how school-based intervention is perceived less stigmatizing than specialized mental health services by both children and their families (Fazel et al., 2016; Nadeau et al., 2017). Second, situating care within the school context enables a contextualizing approach to mental health care, as school-based intervention allows to target social realities that play a major role in shaping refugee children’s development and mental health. Here, studies demonstrate the important role of positive school-based relations (e.g., between peers, student–teacher relations), school policies and positive feelings of school belonging for refugee children’s well-being, their adaption and feelings of belonging to the host-society (e.g., Schachner et al., 2018), while equally indicating how the school operates as social space where refugee children encounter the detrimental developmental and mental health impact of post-migration stressors of social isolation, exclusion, and discrimination (Kia-Keating and Ellis, 2007; Spaas et al., 2021). With scholarly work increasingly pointing to the role of school-based relationships in exacerbating or counteracting the detrimental impact of these post-migration stressors, school-based intervention allows to target these important social variables. Third, school-based mental health care may provide a stepping stone in strengthening parent-school relationships. While refugee parents are often involved and deeply invested in their children’s schooling and well-being (e.g., Roubeni et al., 2015; Bergset, 2017), schools often find it difficult to connect and engage with parents, due to language, cultural and practical barriers, or fear they lack the skills to do so (e.g., Georgis et al., 2014). School-based mental health care could serve to strengthen school-family interactions, which can in turn form a bridge to foster refugee families’ participation in the host society (Rousseau et al., 2012; Nadeau et al., 2017). Fourth, engaging mental health professionals in schools allows for expertise-building in school actors, through processes of case-based sharing of knowledge regarding the mental health impact of forced displacement throughout refugee children’s developmental phases (e.g., Papazian-Zohrabian et al., 2020). In schools, these processes of case-based expertise-building can, for example, support school actors’ future assessment of refugee children’s development, foster adequate signal detection and referral, as well as trauma-informed teaching practices and educational practices in second language acquisition.

Collaborative mental health care, a specific form of psychosocial care provision developed in Canada as a systemic approach in mental health care provisions for immigrant and deprived communities, is an innovative approach in which specialized mental health care is embedded within low-threshold community or primary care settings (e.g., community health centers, schools, daycare centers). In these primary care settings, an interdisciplinary (and often inter-ethnic) support network of professionals surrounding a child and family is set-up and coordinated (Rousseau et al., 2012). This embedding of an interdisciplinary care network within primary care settings aims to serve a three-fold objective (Chenven, 2010). First, collaborative mental health care aims to increase the availability and accessibility of mental health care service provision to minority groups (Rousseau and Guzder, 2008; Ellis et al., 2013). Second, collaborative mental health care sets out to enable a dialogue in which parents and professionals have equal voice (Rousseau et al., 2012; Nadeau et al., 2014). Third, collaborative mental health care aims to empower and foster agency in minority groups within mental health care trajectories, strengthening minority group members’ position and voice within institutionalized health care practices (e.g., Nadeau et al., 2014, 2017).

When implemented in school contexts, school-based collaborative mental health care involves the formation of an interdisciplinary care network, in which mental health care professionals collaborate with school partners, family members and other relevant professionals to engage in a process of joint assessment of child development and mental health, as well as joint intervention planning and provision (Rousseau et al., 2012). Within a collaborative care network, assessment and intervention planning aims at developing an understanding of children’s psychosocial and school-related functioning against the background of families’ migration histories, cultural identifications, and current stressors, while equally connecting to resilience, resources, strengths and hope in the family (Nadeau et al., 2017; Papazian-Zohrabian et al., 2020). In addition to the strong emphasis on the involvement of parents in collaborative mental health care, interpreters or cultural brokers are often key figures in collaborative care network meetings (e.g., Nadeau and Measham, 2006; Georgis et al., 2014; Nadeau et al., 2014). This collaboration with interpreters or cultural brokers has the potential to strengthen the refugee family’s position within the care network, and enables a culturally valid assessment in considering the role of cultural meaning systems in shaping symptomatology and coping strategies (e.g., Brar-Josan and Yohani, 2019).

In this article, we explore implementation of school-based collaborative care for refugee children in primary education and secondary reception education (OKAN), and their families within the Belgian educational landscape. In Belgium, in 2020, 16,910 persons applied for international protection. This number was lower than the year before, when 27,742 persons submitted a request, and the lowest number since 2008. This decrease in asylum applications was related to the outbreak of the COVID pandemic in Belgium in the spring of 2020, the limits it posed for international mobility and the temporary suspension of registering possibilities by the Belgian government in March and April 2020 (Office of the Commissioner General for Refugees and Stateless Persons, 2021a). In 2020, 4,588 persons were granted protection status within Belgian borders, their main countries of origin being Syria, Eritrea, Afghanistan, Turkey, and Somalia (Office of the Commissioner General for Refugees and Stateless Persons, 2021a). Over the past months, starting in the summer of 2021, application numbers have been sharply on the rise, with a recent high point in September 2021 (Office of the Commissioner General for Refugees and Stateless Persons, 2021b). That month alone, 3326 people applied for international protection, one third of them originating from Afghanistan, following the capturing of Kabul by the Taliban in August 2021. Today, November 2021, 26,647 persons reside within Belgian asylum centers, awaiting a decision to their demand for international protection. Almost half these persons form families with children (38%) or are unaccompanied refugee minors (10%) (Fedasil, 2021). The city of Leuven, in Belgium, is located in proximity of several such asylum centers and is home to a substantial number of schools providing (reception) education for refugee children and adolescents. In keeping with a broader national political framework, whereby persons who are granted asylum in our country are obliged to enter into an official integration program that primarily focuses on the cultural adaptation of newcomers in the host country, also the system of reception education for young newcomers is mostly oriented toward promoting their integration. Pertaining to reception education, there exists large policy emphasis on host country language acquisition, but to date little policy attention for working on psychosocial well-being in the classroom (Kevers and De Haene, 2013; Pulinx et al., 2017). Next to a substantial number of reception schools, Leuven also houses the oldest university in Belgium, KU Leuven, where, at PraxisP, the Clinical Centre of the Faculty of Psychology and Educational Sciences, the Transcultural Trauma Care Team develops and provides outpatient care for refugee families. This care derives from an integrative approach rooted in family systems therapy, transcultural psychiatry, and refugee trauma care, with a particular focus on mobilizing family and community relationships in coping with the relational impact of forced displacement and exile and in shaping post-trauma reconstruction (e.g., De Haene et al., 2018; Kevers and Rober, 2020). In a project within the larger frame of the Education Council (Samen Onderwijs Maken; SOM) of the Leuven municipality, an initial project (January 2018–June 2020) set up school-based collaborative networks for the integrative provision of academic and psychosocial support to refugee children, resulting in the operational provision of school-based collaborative care for refugee children and their families as integral part of the municipal policy in support of refugees’ social integration. Under the umbrella of the Education Council, a cooperation between the Transcultural Trauma Care Team for Refugees (PraxisP, KU Leuven) and the Centre for Language and Education (Centrum voor Taal & Onderwijs; CTO) was set up. In close collaboration with complementary regional partner organizations (i.e., Dienst Diversiteit Stad Leuven, Centrum voor Algemeen Welzijnswerk Oost-Brabant, Centra voor Leerlingenbegeleiding VCLB & GO! CLB, and Lokaal Overlegplatform Leuven), we established the interdisciplinary Consultation team for Collaborative Refugee Care (CCRC; Steunteam Vluchtelingen). The Consultation team for Collaborative Refugee Care consists of a consultant in multilingual development (CTO KU Leuven) and a consultant in transcultural trauma therapy for refugee families (PraxisP KU Leuven), together implementing and further developing the practice of collaborative mental health care within schools in Leuven, Belgium. Here, the CCRC realizes an innovative integration of support for refugee children’s academic, multilingual and psychosocial development within a collaborative network of interdisciplinary professionals, school actors, family and community members. In this article, we aim at developing an in-depth description of the collaborative mental health care intervention and to explore potential working mechanisms through case analysis, as initial steps in generating an evidence-base on this novel intervention practice (Veerman and van Yperen, 2007; van Yperen et al., 2017).

## Materials and Methods

This article aims to engage in an initial exploration of the effectiveness of our school-based collaborative mental health care practice. We rely on van Yperen et al.’s (2017) four-stage model for establishing evidence-based youth care to examine the effectiveness of newly emerging intervention practices, recommending specific evaluation methods in each stage of intervention development. In a first stage of intervention development, evidence of effectiveness requires specification of the intervention’s core elements through, for example, descriptive research methods, document analysis and implementation studies. In a second stage, the developing intervention’s underlying theory should be made explicit, relying on literature reviews, qualitative data collection or expert consultation. Gathering evidence on intervention effectiveness in a third stage involves research methods such as pre- and post-test studies, quality control studies and quasi-experimental studies that allow to establish preliminary evidence that the invention works as it is supposed to work. In a fourth and final stage, the intervention is further evaluated through the use of even more rigorous research methods, relying, for example, on randomized-controlled trial studies (Veerman and van Yperen, 2007). For the propose of this article, we relate to the first level of van Yperen and colleagues’ model, working toward an in-depth description of our developing intervention in two subsequent steps.

First, we develop a detailed description of our practice, based on the exploration of case documents, written process reflections, intervision, and an initial identification of intervention themes. This descriptive exploration of our practice was initially developed by the last author and subsequently reflected and commented on by the first and second author. Throughout, the first and last author strived to embed this intervention description within exiting scholarly literature through articulating its interconnections with scholarly work on transcultural and systemic refugee (trauma) care.

Second, we describe an in-depth exploration of intervention processes and working mechanisms, obtained through clinical case analysis of case work collected through our practice in schools in Leuven, Belgium. Analysis of each presented case was initiated by the author involved as a leading clinician within that case, with the second and last author taking the lead in analyzing cases one and three, and the first author developing first analyses of case two. Following this first round of clinical case analysis identifying processes and working mechanisms, the second and last author added their interpretations to the first analysis of case number two, and the first author did the same for the other two cases. In a third and final step, interpretations were confronted, discussed and as such brought to consensus.

## School-Based Collaborative Mental Health Care: An Intervention Description of the Leuven Implementation

### Initiating Collaborative Mental Health Care: Establishing Collaboration and Trust in a Collaborative Care Network

The starting point of a collaborative mental health care trajectory in school are typically concerns about a refugee child’s development, in terms of, for example, concentration difficulties or withdrawn behavior in the classroom, or difficulties in second language acquisition or broader language development. In most cases, collaborative mental health care trajectories are initiated by school partners, expressing concerns about a child’s school-related functioning. However, it is equally possible for collaborative mental health care trajectories to have concerns voiced by refugee children, their families or important others in the child’s or family’s network as a starting point.

When school partners wish to initiate a collaborative mental health care trajectory, in a first phase, explicit consent of parents in setting up a collaborative care network is sought. Supported by a leaflet in six mother tongues, referring actors provide information regarding the collaborative care provision to refugee parents and explore their willingness to start up a trajectory. Parental consent and the negotiation of consent are seen as fundamental to shaping care partnerships based on the principles of equality and agency and need explicit attention and time: parents may experience distrust and show reluctance in engaging with the invitation, often resonating anxieties regarding difficulties in their children’s school trajectories or broader patterns of distrust in host society institutions. Throughout this process, the CCRC most often provides supervision to school actors in how to sensitively communicate to parents about developmental concerns in the child and carefully listen and validate parents’ concerns.

Once parental consent is ensured and based on a preliminary exploration of developmental concerns in referring partners, the CCRC initiates the formation of a collaborative care network including school partners, school counselors (CLB), the child, the child’s parents and other potentially meaningful partners (e.g., social workers), mostly complemented by an interpreter or cultural broker. Once the collaborative care network is formed, the network partners meet with each other in one or in consecutive collaborative care network meetings. The collaborative network’s composition may shift during the course of the trajectory, corresponding to developments in the family’s life conditions or familial developmental phases, or following parents’ expressions of supportive figures within their network. The process of dialogue and alignment between network partners is monitored and facilitated by the CCRC members; network dialogues may at times be complemented with intermediate meetings with parents or school actors separately, for example, if parents request to address family migration history with the CCRC first before sharing with the broader network.

In an initial phase, collaborative network meetings are focused on establishing trust between the CCRC, parents, and all professional partners participating in the network. Validation of parents’ protection and care is central to this process of establishing trust. The CCRC validates, for example, parents’ care for their child’s well-being and schooling, and voices recognition for how the child’s school trajectory may be experienced as an important vehicle in giving meaning to the family’s life-history of forced displacement, and in negotiating social and cultural belonging in the host (Roubeni et al., 2015). Also, the CCRC validates the parental protection and care underlying hesitations parents may voice in engaging with the network (e.g., Rober, 2002), and normalizes symptoms as prototypic responses to forced displacement. Finally, informed consent forms provide all network partners with information and decision-making options pertaining to the treatment and exchange of confidential and personal information by the CRCC during or between the collaborative care meetings. By engaging in a conversation about consent and confidentiality, the CCRC members hope to establish a sense of trust in the refugee family about sharing information between network partners. The commitment of all network partners to the refugee family is made explicit by signing this informed consent.

Following this initial phase, all partners jointly engage in an in-depth assessment of the child’s development and functioning, as well as the co-creation of an intervention plan and a joint delivery of this intervention. Thereby, both assessment and intervention are developed through the lens of a systemic perspective on refugee mental health care (De Haene and Rousseau, 2020).

### Assessment in Collaborative Mental Health Care

**FIGURE 1:**
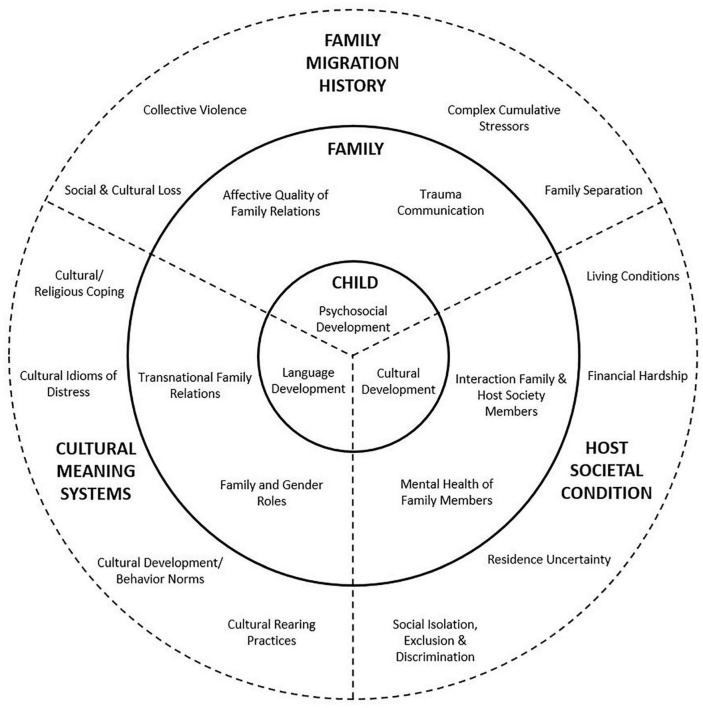
Model for contextual assessment of psychosocial, language, and cultural child development (based on De Haene and Rousseau, 2020).

Through a series of collaborative care network meetings, engaging in a dialogue between all network partners, and potentially through additional assessment, network partners develop an in-depth understanding of child development, in terms of psychosocial, linguistic and cultural development. Hereto, network partners are first invited by the CCRC to share their understanding of the child’s socio-emotional, behavioral, cognitive, and linguistic development and voice their concerns. School actors often start by sharing their experience of the child’s functioning in classroom and peer relationships, and provide an insight into the child’s achievement and progress in school tasks, often including a focus on host society language acquisition. Building from this sharing of concerns, the network discussion aims at broadening an individualizing (and mostly problem-oriented) depiction of child functioning toward a shared systemic perspective on refugee child development, through an exploration and contextualization of child development in relation to three dimensions: cultural meaning systems, the refugee family’s migration history, and the family’s social condition in the host society (e.g., Measham et al., 2014; De Haene and Rousseau, 2020; Spaas et al., 2022). Hereby, these three dimensions are considered not only in their role in shaping child development, but equally in how they encroach upon family relations, which provide the primary context for child development, mental health and adjustment in the aftermath of forced migration (De Haene et al., 2013; Guzder, 2014; De Haene and Rousseau, 2020). Figure 1 (Based on: De Haene and Rousseau, 2020) provides an overview of this assessment approach. This model visualizes how child development is embedded within family relationships and its dynamics, with the family’s migration history, cultural meaning systems, and current social conditions encroaching upon family functioning. Here, family relationships are structured by coping with and giving meaning to family migration history, host society conditions, and cultural belonging in exile.

First, assessment within the collaborative network mobilizes an exploration of cultural meaning systems at play in refugee parents’ understanding of their child’s development and functioning. As culture shapes child rearing practices, normative notions of developmental trajectories and goals, and patterns of interaction and communication between parents and their children (e.g., Durgel et al., 2013; Otto, 2014), valid transcultural assessment implies taking these parameters into account and inviting parents to talk/share about cultural patterns and beliefs in parent-child relationships and child development. This focus extends to exploring, most often supported by the cultural broker, cultural idioms of distress, explanatory models, and coping strategies mobilized by the refugee family, their extended family network, or community members, in their lived experience of distress, developmental concerns, or illness (e.g., Lewis-Fernandez et al., 2016). This exploration includes an interest into how the refugee family negotiates complex processes of cultural change and continuity in living through exile. School actors are invited to share their understanding of the child’s developing cultural belonging and identifications within the school context, as a starting point for a shared exploration with refugee parents on how the child’s school trajectory may be embodying the encounter between home and host society cultural meaning systems and behavioral patterns (e.g., Schachner et al., 2018). Further, network discussions may address differential acculturation patterns between parents and children, explore parental lived experiences or migration-related changes in disciplining strategies and authority in interacting with other socialization actors such as the school, or invite dialogue on experiences of cultural loss and uprooting experienced by family members. Network discussions thus include an open exploration of meaning-making of cultural continuity, cultural change, and hybridity within family relationships and within the child’s school trajectory, with a particular interest into how these dynamics may be at stake in shaping child development, symptom interpretations, or coping strategies. For example, very often, gender role changes (such as the exile-invoked loss of the paternal roles of protective figure ensuring economic provision of the family) are brought into dialogue within the network, and provide a space for containing cultural bereavement and validating parental strategies of providing protection and care (e.g., Guzder, 2020).

Second, a core focus of network discussions during the assessment phase is the exploration of how the family history of forced migration and traumatization encroaches upon family development in exile. Within the network, parents are sensitively invited to bring in those parts of their family history they wish to share. Respectfully supporting parents in sharing fragments of their (transgenerational and transnational) life-histories takes place through inquiring into how parents experience the onset of symptomatic behavior or the impact of their migration trajectory on presenting symptomatology. Here, parents are often invited to recount the child’s school trajectory during pre-flight and flight phases, supported by the CCRC through psycho-educative and normalizing accounts of prototypical sequelae of forced displacement. Here, tentatively exploring the families’ experiences, using sentences such as “what some parents have told us”, as it simultaneously allows for a normalization of symptoms and a careful, attuned approaching of potentially traumatic life experiences often proves to open dialogical space. In this process, school actors’ expression of empathy and solidarity with the family’s plight often plays an important role in establishing a supportive dialogical context. In the course of this exploration, network discussions may explore a myriad of traumatic stressors, such as social and political instability, experiences of war and violence, and the loss of community and family members (Hodes, 2000; Lustig et al., 2004), as well as experiences of deprivation and exploitation during the flight phase or in temporary residence (e.g., Arsenijević et al., 2017). Very often, experiences of family separation surface. In refugee families, family separation is a highly prevalent dynamic, in which children are separated from their parents or other important caregivers, either due to financial reasons in arranging for all family members’ flight, or unexpectedly by smugglers during their flight. Family separation often lasts for a prolonged period of time with uncertain timing and outcome of reunification (e.g., Rousseau et al., 2001; Miller et al., 2018). Here, an explicit focus is to develop an understanding of how experiences of family separation (and ongoing efforts toward family reunification) have shaped past and current family interactions (such as parental traumatic expectations or overprotection, or gender role changes), while equally holding the often strong accounts of anxiety, bereavement, guilt and sense of failure to protect expressed by refugee parents regarding family separation.

Throughout this identification of migration-related stressors and their meaning within family relationships, an explicit focus of exploration lies on how this cluster of cumulative traumatic stressors encroaches upon family functioning. This necessitates exploring and connecting to the specific mode of trauma communication adhered to within the family (e.g., De Haene et al., 2018). Many refugee parents emphasize their pattern of active silencing of the traumatic past within family relationships, and may voice hesitation on an open sharing regarding family migration history with their child. This parental silencing strategy might be oriented at avoiding a reactivation of distress or harm in their children or themselves (e.g., Dalgaard and Montgomery, 2015; Dalgaard et al., 2016; De Haene et al., 2018). Validating parental protection and care, and voicing the network’s engagement to carefully align with parents’ orientations on finding secure ways of sharing on traumatic history without breaching their orientation on avoiding reactivation of distress or further harm in their children or themselves, may often provide a starting point for further engaging parents in an exploration of intra-family sequelae of forced migration stressors. Relatedly, an important dimension of exploration concerns parents’ mental health and their availability to provide dyadic safety within parent-child attachment relationships. Provoked by traumatic responses, refugee parents may display overprotection or avoidance, or develop shared intrusion within the parent–child dyad when confronted with the child’s anxiety or hyperarousal (e.g., Almqvist and Broberg, 2003; De Haene et al., 2010, 2013). To develop an understanding of these potential dynamics in which traumatic response becomes inscribed within parent-child interaction, parents are invited to explore their child’s symptomatic functioning within the family context, to account for their strategies or concerns in coping with their child’s distress, and to address potential changes within dyadic interaction invoked by (traumatic) stressors and loss experiences. Furthermore, specific family processes of gender role changes (e.g., Hodes and Hussein, 2020) or the potential increase of intra-family stress or conflict (or intra-familial violence) (e.g., James, 2010) are brought into conversation, providing a validating stance through externalizing the sources of unsafety within broader migration-related social conditions. Further, network dialogues emphasize an exploration of how refugee family relationships are a key locus for meaning-making in the aftermath of traumatization. Here, exploring the meaning of the child’s school trajectory in parental orientations on restoring future perspectives in exile generates an open dialogue on post-trauma reconstruction of meaning, where parents may address their understanding of the child’s school-related development as a vehicle of restoring hope and justice (Cissé et al., 2020). Equally, the network dialogue may address how family members mobilize cultural belonging in shaping a meaningful engagement with traumatic suffering (e.g., Kevers and Rober, 2020), generating a shared understanding within the network on how, for example, behavioral patterns of religious practices operate as a vehicle of establishing continuity in the face of loss or how the child’s host country language acquisition is lived as both a reiteration of cultural loss and bridge toward a meaningful future.

Third, assessment within our collaborative mental health care practice contextualizes child development within the refugee families’ social condition in the host country. With existing research underscoring the role of post-migration stressors (e.g., financial stressors, prolonged residence insecurity, inadequate housing, social isolation, ostracism and social exclusion) in maintaining or aggravating the negative impact of traumatization, loss, and exile on mental health and development in refugee children and their families (e.g., Vervliet et al., 2014; Keles et al., 2016; Kohli and Kaukko, 2017; Miller and Rasmussen, 2017; Spaas et al., 2021), assessment within the collaborative networks holds an explicit emphasis on locating the child’s and family’s development within these broader social conditions. Hereto, the CCRC explores the family’s position within their broader social fabric and cultural community, and invites parental accounts of potential experiences of material stress, socio-economic deprivation or experiences of racism and discrimination. Of particular interest in this exploration is supporting a shared understanding of how the refugee family’s social condition is echoed within family-school interactions. Here, family experiences of social isolation or discrimination are not merely addressed in their impact on family relationships, but equally in how they might resonate in parental distrust or strategies of avoiding or withdrawing from communication with school actors (Benoit et al., 2008), in school actors’ representations of refugees’ parenting skills mirroring or counteracting broader, often deficit-oriented stereotypes on immigrant parents (De Haene and Rousseau, 2020), or in dynamics of traumatic reenactment between the family and the school as a representative of host society institutions (Rousseau, 2020; Spaas et al., 2022). Equally, network partners engage in an exploration of the way broader social dynamics may encroach upon the child’s acculturative tasks within the classroom and school, addressing the role of teacher-pupil and peer relationships and scrutinizing potential dynamics of exclusion impacting the child’s psychosocial and linguistic development (Evans et al., 2020).

Assessment in collaborative mental health care thus constitutes a joint process of contextualization of child development within family relations and family development, and within the dimensions of cultural meaning making systems, the refugee family’s migration history and their social condition in the host society. This assessment informs the process of collaborative intervention planning.

### Intervention in Collaborative Mental Health Care

Intervention planning in school-based collaborative mental health care includes interventive processes on four different levels: interaction between school actors and the refugee family, classroom practice, school policy, and the mobilization of external social care provisions or referral to specialized mental health care services. Intervention planning in collaborative mental health care is equally rooted within systemic perspectives on refugee mental health care, aiming to move beyond an individualized approach, toward targeting social realities that play a major role in shaping refugee children’s development and mental health, and to explicitly mobilize the relational encounter between the refugee family, school partners, and potentially meaningful others in the collaborative care network as a microcosm of these social realities.

First, on the level of school-family interactions, establishing trust and positive collaborations between school actors and the refugee family is a central dimension of collaborative intervention, often woven into the assessment phase (Nadeau et al., 2017). With the refugee family, we build trust through validating parental protectiveness, care, and hope for future perspectives, welcoming minority perspectives (including the use of mother tongue) in the school as a representative of the majority group, and stigma-reduction in validating and normalizing child behavior. With school and other professional partners, validating their commitment and challenging existing stereotypes on refugee families form a central aspect of fostering trust within school-family interactions. This supporting of trustful, secure partnerships between school actors and the refugee family is inscribed within a broader interventive goal of stabilization in post-trauma reconstruction, where the regaining of control and adequate coping strategies operates as a core reparative strategy (e.g., Herman, 1992; Van der Kolk, 2014), and an important precondition for positive child development in general. Further on the level of school–family interactions, collaborative care intervention may entail the negotiation of cultural differences in child rearing practices between the parents and school actors, and to establish partnerships between refugee parents and school actors in which different cultural practices of disciplining, monitoring, authority, and proximity are validated. Finally, psycho-education with both the refugee family and school partners often takes place as part of interventive steps. Psycho-education can, for example, be directed at explaining the impact of collective violence, traumatization or family separation on family development as well as on the child’s psychosocial functioning and language acquisition. At times, it may be valuable to exchange the setting of the collaborative care network meeting for separate meetings with either school partners or the refugee family. These meetings can allow for an engagement with content that is otherwise too difficult to address in the larger collaborative care network, or entail a further engagement with school actors on sensitive issues shared in collaborative network meetings. During these separate encounters, partners negotiate consent about how to share about this intermediate meeting within the broader network.

Second, on the level of the classroom, intervention may include the sharing of expertise in specific teaching practices for young refugee newcomers, or provide relevant learning aids to refugee children. Intervention can be aimed at supporting (second) language development, as well as at fostering refugee children’s psychosocial development and mental health, through mobilizing social support within the classroom (e.g., promoting and supporting positive peer and teacher-pupil relations, promoting a positive, safe and welcoming classroom atmosphere). For example, this may refer to strategies of providing structure and mentalizing co-regulation for refugee children displaying arousal or aggressive problem behavior, creating spaces for containment of missing for a bereaved child within the teacher–child interaction, or jointly designing strategies to welcome the child’s cultural background within classroom communication. Within the collaborative care network meetings, another meaningful form of intervention at the classroom level consists of insight- and expertise-building in teachers through collaboration with the CCRC (e.g., Papazian-Zohrabian et al., 2020). This might allow teachers to better understand and support the refugee child and family, and other refugee children and families over time.

Third, joint intervention planning within the collaborative care network can address school team functioning and policy practices. Here, case-based joint intervention planning and expertise-building can bring forth adapted school policies, for example, with regard to strategies for the involvement of refugee parents in schooling trajectories, previously established teaching practices with refugee children, or the school’s diversity policies (Celeste et al., 2019). Furthermore, the collaborative meetings are often interwoven with an ongoing supervision of school teams, focusing on the containment of insecurities, feelings of powerlessness or indignation often experienced by school actors while engaging in the network meetings, or addressing potential splitting processes or institutional reenactment within the school team. Here, the CCRC provides holding of the affective responses, while mentalizing these processes of prototypic relational dynamics in working with traumatized populations.

Fourth, outside of the school context, collaborative intervention can mobilize external social care provisions in supporting the refugee child and family. Also, when all partners of a collaborative care network deem it necessary, refugee children and their families can be referred to specialized mental health facilities (Rousseau et al., 2012). Among other, such referrals can be directed at realizing specialized diagnostic assessment of refugee children’s (linguistic) development and mental health, therapeutic support in the areas of language acquisition or child mental health, or transcultural trauma therapy for the refugee family. Table 1, below, provides an overview of the different contextual levels in which we develop interventions in collaborative mental health care practice.

**TABLE 1:** Intervention in collaborative mental health care.

Intervention level	Possible interventions
School–family interaction	Trust building (validating commitment, normalizing behavior, challenging stereotypes) Witnessing of the refugee family’s life story Psycho-education Negotiation of cultural child rearing practices and expectations
Classroom	Support of teaching practices Provision of learning aids to the refugee child Mobilization of social network and support in the classroom
School-Policy	Case-based expertise building Adaptation of school policies (e.g., parental involvement, teaching or school diversity policies)
Referral to external care services	Referral to external social care services Referral for specialized diagnostic assessment of development Referral for specialized (individual/family) therapy

## A Further Exploration of Processes and Working Mechanisms Through Clinical Casework

We now further engage in an in-depth exploration of processes and working mechanisms of our collaborative mental health care practice. Below, we present three cases^1^, after each of which we explore processes and working mechanisms through a reflection on the development of an assessment and of interventions in the presented cases.

### Amina—Can We Bridge Cultural Worlds in School?

A primary school contacted the CCRC (the second and the last author) concerning Amina, a 9-year-old girl from Eritrea, the eldest child of four. Amina settled in Belgium 2,5 years earlier and is now in second grade. In their referral, school actors voiced their concerns about Amina’s behavior on the playground, including daily conflicts with peers, angry outbursts with physical aggression, and difficulties following the school rules. In the school team’s experience, Amina often seemed disconnected from social interactions within the classroom and school, and unable to adequately relate within peer relationships. The school expressed their orientation on developing a better understanding of Amina’s behavior, in order to support her appropriately. Following the CCRC’s proposal to initiate a collaborative trajectory with Amina’s parents, the school team shared their sense of multiple thresholds in parents’ involvement in parent-school interaction. Here, an initial collaboration between the school team and CCRC focused on transmitting knowledge on how cultural notions on parental responsibilities and school authority may shape parents’ withdrawal from active dialogue with school actors, and how sharing and validating these cultural differences may be important in understanding possible barriers for parents to participate in school events. With the help of the CCRC and a cultural broker, the school engaged in a careful process of inviting Amina’s parents for an initial collaborative care network in the school.

Amina’s father arrived alone at the first collaborative care network meeting. Following the provision of information by the CRCC and school team, Amina’s father emphasized not feeling familiar with the concerns about Amina’s relational behavior in the school context, but shared the school team’s request to understand Amina’s behavior. Here, the CCRC introduced the interest in exploring the potential role of Amina’s and her family’s migration trajectory in shaping her current distress. Father openly shared fragments of the family forced displacement, recounting how his children were too young at the time of his political problems to suffer from them and briefly referring to a year-long family separation. When the CCRC-consultant voiced how separation, but equally reunification are often highly stressful phases, Amina’s father nodded intensely. He took his mobile phone and became emotional while he walked around the circle of network participants, showing pictures of a playing and smiling Amina in Eritrea. The consultant provided holding and mentalization, in telling father how she resonated with father’s hope his children would not be irreparably marked by the family’s history of forced displacement, and how not speaking about the past may feel protective in preventing further harm. At the end of this meeting, father expressed his wish to meet again and jointly explore how the family migration history may be impacting his daughter’s development.

In subsequent collaborative network meetings, father, the school team, and the CCRC joined in a further, contextualizing understanding of Amina’s relational disconnection and dysregulation. Here, the network developed several perspectives potentially at play in Amina’s relational functioning.

First, the collaborative network aimed at understanding potential interactions between Amina’s relational functioning and her ongoing development in language acquisition. Hereto, network partners shared information on Amina’s seemingly very limited vocabulary and linguistic interaction with others in the school context, complemented by father’s sharing of his observation of differential mother tongue use among the siblings. Father also recounted Amina’s stories of not being able to ask the teacher for explanations nor talk to the teacher when she had been bullied. It became apparent that Amina’s relational functioning may be closely intertwined with this seeming stagnation or delay in language acquisition, with language difficulties evoking frustrated or overwhelmed behavior in Amina and rendering physical violence her first response in conflicts. In further exploring this hypothesis, the CCRC consultant in multilingual development and education carried out a classroom observation and provided Amina’s teacher with specific didactic methods to support classroom practices that allowed for maximizing Amina’s learning trajectory. Further, the collaborative network facilitated access to diagnosis and remediation by a speech therapist.

Second, systemic processes of acculturative tasks within school–family interaction were explored as potentially relevant in understanding Amina’s relational dysregulation and coercive behavior. This focus emerged after the school team ostensibly showed incomprehension and frustration with father, who did not explicitly seek partnership with the school team in co-regulating Amina’s difficult behavior. Father explicitly expressed his inability to modify Amina’s school-related behavior and transferred his clear focus on authority to the school actors. Mobilizing in-depth information provided by the cultural broker during an intermediate conversation, the consultant invited father to talk about how, within his upbringing, family-school interactions were performed. Vividly, father recounted how respecting the school’s authority was a central family value, where the teacher was respected in his/her role as an important agent of moral socialization and where teacher discipline very often involved physical punishments. Father further expressed how by remaining at a distance from family–school interaction, he aimed to show great respect for the autonomy and authority of the school: he confided in the school’s disciplining, and it felt very inappropriate for him to interfere as a parent. As father’s account expressed an understanding of parental involvement at odds with the school’s expectations, the consultant voiced how the network seemed to encounter relevant cultural differences in understanding normative parental tasks. This locating of parental behavior in cultural practice counteracted stereotypical representations of Amina’s parents within the school team, and invited a further exploration of cultural notions of discipline and parental authority. Father recounted his experience of cultural differences in disciplining strategies, referring to the emphasis on caregiver authority and strict discipline in the family’s home country that was now confronted with an emphasis on participation and positive reinforcement. Through holding father’s sense of loss in practices of authority, we enacted in the room how Amina was daily crossing a bridge between different cultural worlds, walking between father and the school’s care coordinator. Amina’s father resonated strongly with this enactment. At the end of this meeting, partners within the care network agreed that it may be supportive to Amina if adults assisted her in crossing this bridge, and a follow-up meeting was planned to develop a shared understanding of authority and disciplinary practices. Throughout these subsequent meetings, locating child and family functioning within socio-economic conditions operated as another important emphasis. At the beginning of our series of network meetings, the family experienced a lot of stress in terms of housing and finances. Throughout the meetings, we saw the family becoming more relaxed as they found more suitable accommodation and new job possibilities. The relief this brought in the family had an effect on Amina, who was able to find more peace at home as a result.

Amina’s story illustrates how the collaborative care process starts in the case that a referring school expresses difficulties in finding ways to connect with the parents. As described earlier, refugee children’s parents are often deeply concerned about their children’s schooling and well-being, but at the same time show hesitation toward parental participation at school. It is important to note that such hesitations are often also present among school partners, as was the case in Amina’s story, where school partners experienced communication/language barriers and feelings of incompetence in relating to Amina’s father. Here, the school was supported by the CCRC to connect with Amina’s father and to facilitate network meetings, together with a cultural broker. Together with the cultural broker, the CCRC facilitated a dialogue within this network about cultural notions of child rearing practices, parental participation, developmental tasks and milestones. This intercultural dialogue made it possible to contextualize Amina’s development within the ongoing dynamics of cultural change and belonging to minority culture. Further, the collaborative care network meetings enabled an understanding of Amina’s behavior within current stressors and how she was not able to give words to and voice these stressors, her behavior embodying a lot of the stress and powerless she encountered at home. Further work with Amina’s family was oriented at locating Amina’s behavioral and language difficulties within the context of the family’s migration story. Indeed, in addition to developmental factors, language difficulties may express children’s and families’ coping with traumatic migration history or bereavement. Indeed, at the end of our first meeting, Amina’s father told the network partners, in little words, how not talking about their past provided him with a way to protect his children from reliving pain or intrusive memories.

This case furthermore illustrates the complexity and interconnectedness of the differential diagnostic process in the transcultural assessment of language difficulties. Interconnected with the exploration of anamnestic information about language acquisition in mother tongue and the host society’s majority language, transcultural assessment tries to understand this acquisition process within the context of existing stressors and within the context of the migration history and different coping strategies. In Amina’s case, transcultural and systemic assessment resulted in a shared plan for intervention focused on bridging cultural differences by facilitating a dialogue between key partners on the one hand and supporting language skills on the other, both individually, at the classroom level and at the school policy level. By working together on an optimized (second) language acquisition policy at school and by increasing learning opportunities inside and outside the classroom, the CCRC aimed further at supporting the school as a central developmental setting.

### Ahmed—Can We Restore Safety in School?

Ahmed was a thirteen year old Iraqi boy, the youngest in a Muslim family of six children. His father, the head of the family, was a carpenter in Iraq. Ahmed was referred to us (the first author) by his teacher and the student counseling service (CLB), describing concentration difficulties in class, hyperactive behavior and externalizing behavioral difficulties, whereby Ahmed frequently interrupted lessons and got into conflicts with his peers. The school team and CLB referred Ahmed for diagnostic assessment, suspecting ADHD. They informed us that Ahmed’s parents knew of the referral and had given their consent for a potential diagnostic trajectory.

We first met Ahmed and his mother at a school meeting. Soon after we sat down, we realized that Ahmed and his mother did not understand why a child psychologist (the first author) was present at school. Ahmed’s mother reacted fearfully, her fear seemingly touching upon cultural perceptions of mental illness and perhaps mental health stigma, as she described how she saw referral to a psychologist meant that the school team believed her son to be crazy. School partners answered by explaining their reasons for inviting a psychologist and stressing their concerns about Ahmed’s behavior in class, as well as their ADHD-hypothesis. Left with little room to negotiate her presence with the family, the psychologist instead tried to focus on the mother’s hesitations, their meaning and mother’s own concerns for her son. Ahmed’s mother responded by talking about the family’s migration history, including a lengthy period of family separation. She told us that her eldest son fled Iraq first and that the rest of the family joined him only much later. She emphasized the fear and pain felt by all family members at the time, and how they still often fear losing each again. Thereby, she remarked how Ahmed’s difficulties in school seem to have started at the very moment that his brother, who used to be in Ahmed’s class, switched classes. She also described the many ways in which she still fears for the safety of her children. While recognizing that her son could probably benefit from some extracurricular physical activity to get rid of build-up energy, she told us that she is too afraid to let him out of her sight and therefore prefers to keep him in sight, at home and close to her. At the end of this meeting, we summarized a number of concerns shared by Ahmed’s mother and school partners. We subsequently decided to engage in a broad diagnostic assessment of Ahmed’s development, taking into account the family’s migration history, and the individual and relational impact of the traumatic experiences associated with it.

Following this first meeting, we met separately twice with Ahmed and his father, within the context of our clinical center. Like in school, the first meeting at our clinical center was characterized by palpable hesitations, on part of both Ahmed and his father. Ahmed’s father commented on the concerns voiced by the school team: “I don’t find it problematic that my son does not sit still in class, I didn’t teach him to do so. In Iraq, parents don’t teach their children to sit up tall like the Arabic letter ‘I’, as seems to be demanded of children in this society.” Questions (carefully) probing into the family history were answered evasively. Ahmed’s father told us that he did not wish to speak about the past, but instead wanted his children to forget about Iraq, about the war and what their family had to live through. Perhaps, we reflected, Ahmed’s externalizing behavioral difficulties then formed a language to talk about his fears and pain, without burdening his father with accounts of the past and confirming his father’s fear of having been unable to protect him, his brothers and sisters.

The next time we saw Ahmed and his father, our therapeutic alliance with them felt improved. A while into the conversation, Ahmed’s father told us that he and his wife did not share the school’s concerns and also did not agree with their referral of Ahmed to our clinical center. He said that earlier he had believed it impossible to voice these opinions within the school context and our clinical center, afraid to jeopardize the family’s safe legal position in the host country: “Being a refugee in your country, I should probably stay silent and do what is asked of me by your institutions. That is the only way for me to ensure and protect my children’s future.” Thereupon, Ahmed’s father started talking about a recent incident at school. He told us that Ahmed was seen by a doctor at school, without the family having been notified. According to father, Ahmed was very scared at the time and tried to refuse seeing this doctor. He then asked for his brother to accompany him, but his brother appeared nowhere nearby. Father continued by stressing how the incident affected not just his son, but himself and his wife as well. Before, the school had been a trusted partner in the care for their children, but since the incident parents felt distrustful toward the school partners. He said they no longer thought of the school context as a safe place for their children and wondered if that doctor had been present at school to conduct experiments on their son and other children. Ahmed then told us, quietly, that he also never felt safe in school afterward, his trust in school partners even further diminished by the distrust he sensed in his parents. Later we learned that the doctor’s visit at school formed part of the school’s collective vaccination program, but that this wasn’t clear for Ahmed and his parents. The experience therefore evoked strong feelings of insecurity and fear. This fear further encroached upon the diagnostic trajectory, as it turned out that before our first appointment, father had instructed Ahmed to use the bathroom and run out, should the psychologist invite him into a separate room and attempt to perform experiments on him. In short, school policy on collective vaccination seemed to have been re-traumatizing for this family, triggering a traumatic past of attending school in an unsafe, war-torn country and a painful period of separation during which family members felt unable to protect one another. This unintended, but quite profound re-traumatization in turn elicited dynamics of traumatic reenactment on behalf of the family members, post-traumatic functioning imbuing the relations between Ahmed, his parents and the school team.

Discussing these relational dynamics of traumatization and re-traumatization within the school context seemed a potentially meaningful next step in our trajectory with this family. We therefore set up a new meeting with Ahmed, his parents and the school team. There and with parental consent, we addressed the incident and how what had happened had felt like a repetition of trauma for this family. During the meeting, Ahmed’s father drew explicit connections between the doctor’s visit, it’s impact on the family and Ahmed’s behavior in class (concentration difficulties, agitation, and relational conflicts). He also connected some of the concerns voiced by the school team to the family’s history of living under war: “I taught my son to be hypervigilant, because paying attention to every little detail, noticing every little sound and movement can be of life saving importance in times of war.” Ahmed’s parents then also openly discussed their own concerns about their son, such as the transition to the next school year and the impact of the different stressors the family faced in resettlement on the well-being of their son. By the end of the meeting, Ahmed, his parents and the school team agreed to work together toward providing adequate support for Ahmed in class. We felt the meeting supported all parties present to gain insight into the way relational dynamics of re-traumatization had underpinned Ahmed’s difficult behavior at school. The meeting also seemed to restore trust between the school and family, reuniting them in their care for Ahmed. Lastly, during the meeting, Ahmed’s father openly and repeatedly voiced his ideas and opinions, speaking out where before he felt he should remain silent. We proposed an external referral of the family to a specialized therapeutic practice for systemic, transcultural trauma therapy. In light of the fruitful school meeting, however, parents indicated that they did not currently feel the need for therapeutic family counseling.

Ahmed’s case illustrates several of the central aspects of both assessment and intervention in collaborative mental health care practice. With regard to assessment, network partners engaged in a meaningful contextualization of Ahmed’s restless, defiant behavior and concentration difficulties within the dimensions of cultural meaning systems, the families’ migration history and social condition in resettlement. Pertaining to the dimension of cultural meaning systems, Ahmed’s father, for example, detailed how his cultural perceptions of adaptive child behavior contrasted with the expectation voiced by school-partners that Ahmed would sit completely still in class. A further dimension of contextualization related to the locating of Ahmed’s worrisome behavior within his family’s traumatic forced migration history. Being attentive to patterns of trauma communication in the family indicated that Ahmed’s behavioral symptoms seemed to speak of traumatic suffering that did not tolerate being put into words, in an attempt of Ahmed’s parents to protect their children by silencing traumatic experiences (e.g., Angel et al., 2001). Important reflections were shared on how surviving the war in Iraq altered parents’ child rearing practices, and shaped patterns of interaction and trauma communication within the family. Here, patterns of trauma communication in the family furthermore seemed to shape the clinical encounter and thereby the assessment itself, since answering to the demands of an assessment seemed to oppose Ahmed’s father’s need to silence parts of the family’s trauma narrative. On the level of the family’s traumatic migration history, network discussions served at identifying and containing important relational dynamics of trauma repetition and traumatic reenactment between the refugee family and school partners, actively shaping Ahmed’s development and behavior in the school context. Intervention in Ahmed’s case was mostly located at the level of the interaction between parents and school partners, addressing the relational dynamics of trauma repetition and traumatic reenactment that so strongly impacted Ahmed’s behavior and well-being in the school context. This intervention fostered relational safety between the school and family, opened up new possibilities for dialogue, and seemed to have empowered parents within this dialogue, whereby Ahmed’s father claimed a voice after having felt silenced by our host society before. Ahmed’s case also illustrates how working twice with parents and Ahmed separately allowed for a conversation on issues that were initially too sensitive to address within the school context, or even impossible to address there because of the felt power dynamics between parents and the school team.

### Abdul—Can We Understand the Reiteration of Trauma in School Relationships?

An elementary school contacted us (the second and last author) us in response to their growing concern about the behavioral and moral development of one of their students. Abdul was a twelve-year-old boy who was reunited with his fourteen-year-old sister and mother in Belgium a couple years before, after a prolonged period of family separation. For three years, Abdul and his sister stayed separately with different family members, traveling around Somalia and Ethiopia. Their mother escaped to Belgium without them, without the kids knowing, telling them she was off to work. According to school actors, Abdul’s father had died before mother’s flight to Europe, but the circumstances of his death remained unclear and unspoken about. The school actors strongly valued the academic progress both children were making after never going to school before, but on the other hand, the teachers, school board and the Student Counseling Service (CLB) indicated that they were extremely concerned about Abdul’s behavior toward teachers and other children. They described him as a very dominant and manipulative boy who lied a lot about conflicts with peers. At the end of this initial meeting, we proposed to organize a meeting between the teachers, the school board, the CLB and Abdul’s mother. We explained that the aim of this meeting was two-fold. First, to come to a shared understanding of Abdul’s behavior within the multilayered context of his family, migration and resettlement story and second, to support positive interactions between the family and the school actors.

Gradually, throughout this first meeting, Abdul’s mother shared, with a lot of intense affect, a painful story about a long and painful period of family separation. Her sadness and powerlessness were palpable to everyone when she told that after resettling in Belgium she learned that Abdul was severely abused by his family in Ethiopia, after other family members sent her pictures of the wounds marking Abdul’s body. For several years and without the safety or closeness of his mother, Abdul grew up without caring and predictable adults, in contrast to his sister, who was hosted by another family within the kin and who cared for her protectively. Mother shared her overwhelming anxieties during family separation, and the CCRC-consultant shared her understanding of potential feelings of overwhelming guilt invoked by her son’s abuse and her inability to protect her son. This account invited expressions of recognition by the school actors toward the family, and Abdul’s mother, who may at that point have been valorized in her position of a protective caregiver, openly requested the school not to dismiss her child. During this first meeting, a psycho-educative account of prototypic trauma response in children was shared by the CCRC, The CCRC reflected on how, though the traumatic incidents were situated in the past, daily life and interactions with others may become the space of reliving traumatic life experiences, and minor conflict or tensions may activate a deep sense of injustice, unsafety and fear toward authoritative adults in Abdul.

In a next network meeting with Abdul’s mother, the CRCC (the second and last author, together with consultant multilingual development) further explored how her guilt inhibited her in disciplining Abdul, and how she foremost wanted to give him every opportunity to build a new future in Belgium. In mentalizing the hope underlying the mother’s narrative, Abdul’s mother expressed how a successful school career for her son could form the gateway to a reparative future in the aftermath of war, traumatic separation and abuse experiences, and allow the family to forget their painful past. These first meetings allowed a shift in the way the school actors viewed, understood and connected with Abdul and his family. The network understood why it was difficult for Abdul and his mother to acknowledge and talk about the concerns at school, because it touched upon the feelings of guilt and upon the hope that lies beneath this successful school career. Where Adbul’s behavior was previously understood from lack of guilt, it was acknowledged now as a coping mechanism and a way of protecting the family’s vivid hope to restore the past.

Despite everyone stating that it was a meaningful meeting that brought everyone closer together, a few weeks later we got an alarming phone from the head of the school board to tell us that the situation in the classroom had escalated. They were concerned to such an extent that their last solution seemed an exclusion from the school in combination with child psychiatric or intensive psychotherapeutic counseling. They mentioned that his classmates are afraid of his behavior and that teachers are losing their will to put in the effort. In an intensive process of dialogue with the school, the CCRC aimed at holding fear in school actors while at the same time supporting an understanding of the re-traumatizing impact of this exclusion, not only because this was a repetition of a life history submerged in rejection, injustice and extreme punishment, but also because this might confirm and strengthen an identity as an offender, during an age where he is fully engaged in the developmental task of identity-building. We planned a meeting with school actors to discuss this possible impact of exclusion. At the same time, we tried to direct the family to a parallel outpatient therapeutic trajectory in PraxisP KU Leuven. Partly due to the threat of exclusion, the family agreed to these sessions, but the stigma surrounding mental health care was strong. A male, confidential school counselor from the CLB proposed to participate together with the family, lowering the threshold from the family to participate. During the initial therapy sessions, we get to know Abdul as a very skilled boy. He managed to tell us, in his own words or metaphors, about trauma triggers at school, symptoms of trauma during the night and direct and indirect themes of discrimination and racism. During these stories, both therapists felt the deeply pervaded sense of injustice he is feeling at home and at school. He shared that he was deeply concerned that everyone saw him as the bad guy while his body made him do things that were not under his control. Parallel to the ongoing trajectory, the CCRC sought help from a Somali intercultural broker to contextualize the difficulties within the dimension of cultural meaning systems. By involving a cultural broker, we were able to talk about the role of violence within Somalia, about cultural norms surrounding normative child development and ritual, religious forms of help-seeking behavior inside or outside the Somali community. By subsequently also connecting with cultural and religious practices and coping mechanisms, trust and openness increased throughout the various sessions, allowing mother and Abdul to bring in religious practices as a regulative strategy during therapeutic conversations and enabling a shared dialogue on cultural idioms of distress, in which mother recounted her understanding of Abdul’s symptoms as the presence of devils.

The CCRC intensively collaborated with the school principal, in preparing the meeting with Abdul and his mother to discuss their final conclusion to exclude Abdul from the school. In this preparatory phase, the CCRC and the school collaborated on how to set this dialogue in a way that could minimize the re-traumatizing impact of this exclusion. Central threads within the dialogical position revolved around acknowledging the school’s share in this situation, avoiding splitting between different mental health care or school partners, naming their hope and belief in Abdul, and developing a shared goal in supporting Abdul in healing from the injustices inflicted upon him and counteracting the recurring dynamic in which he would become represented as an offender or failure, rather than as a caring and gifted boy who was coping with the consequences of unjust behaviors of caregivers. Finally, in collaboration with the school, the CCRC succeeded to form a strong and stable network between them, the CLB and the intercultural broker. In the search for a new school, this triad offered the family a thread of continuity in the family’s care network within the host society. Thanks to the school and the CLB, this family could relatively quickly be directed to an outpatient therapeutic trajectory that has since become a family therapeutic trajectory where family dynamics, themes of family separation and reunification and socio-cultural themes related to resettlement are central topics of the sessions.

Although the trajectory of Abdul is not finished yet, it already reflects many central aspects of transcultural assessment and intervention in school based collaborative care. With regard to assessment, the initial collaborative care network meetings with Abdul’s mother resulted in a shared understanding of Abdul’s deviant and manipulative behavior within the aftermath of forced migration, a long and fearful period of family separation and the current societal condition in the host community. In collaboration with a cultural broker, the CCRC was able to engage in an intercultural dialogue about cultural meaning systems about child development, different educational and rearing practices and help-seeking behavior inside or outside the Somali community. With regard to intervention, this case illustrates the importance of preventive mental health care at school for refugees. The moment we met these school partners, the interactions between them and the family were already greatly disrupted and everyone felt immensely powerless in the face of the situation. By facilitating the meetings between the family and the school, a large part of this trajectory was therefore situated at the level of the interactions between the school and family and at the level of the school policy level. It consisted of case-based expertise building in school actors through a process of collaboration and knowledge sharing regarding transcultural assessment and cultural- and trauma sensitive care at school. In this way, collaborative care practice made it possible to avoid a polarization within the family toward the host society. Finally, through this intense collaboration, the family, together with the school actors and the intercultural broker, could successfully be referred to specialized therapeutic care, where the initiated complex processes of assessment and trauma treatment can be continued.

## Discussion

In this article, we developed preliminary evidence on school-based collaborative mental health care in Leuven, Belgium. Relating to the first stage of van Yperen’s four-stage model for establishing evidence-based youth care (2007, 2017), we engaged in an in-depth description of our collaborative mental health care intervention and explored its processes and potential working mechanisms through clinical case analysis.

While our description does not allow for a definite identification of working mechanisms in determining psychosocial, linguistic and cultural developmental outcomes, our descriptive analysis indicates the role of central processes that may operate as working mechanisms in school-based collaborative mental health care, namely: enhancing family–school interactions through contextualizing child development within the family’s coping with migration history and social conditions in relation to cultural notions of adaptation and mental health, mobilizing the family–school relationship as a space of negotiating cultural difference and shaping cultural identifications in diaspora, psycho-educative normalization and validation of parental strategies and trauma response, locating children’s linguistic development in relation to migration stressors, social conditions, and current mental health functioning. Across these interventive dimensions, our analysis primarily points to how collaborative mental health care may mobilize the school and the family–school interaction as a vehicle of restoring safety and stability in the aftermath of cumulative traumatization.

Yet, providing collaborative mental health care in school settings also generates complexities in meeting collaborative mental health care’s central premises of enabling low-threshold access and empowerment through giving voice to vulnerable communities. First, while the school is clearly a primary social context that may provide low-threshold access to refugee families, clinical case work indicates that locating care in the school does not in itself allow to reduce stigma. In refugee families’ migration histories, where children’s school trajectories are intricately intertwined with restoring hope for meaningful future perspectives (e.g., Roubeni et al., 2015), bringing in experts in school to address concerns on children’s development may precisely heighten anxieties in refugee parents and activate stigma regarding mental health. Second, while collaborative mental health care centrally aims at giving voice and strengthening empowerment in vulnerable communities, our practice with refugee families point to the, at times subtle, occurrence of dynamics of reiterating power inequalities between refugee families and majority group members, such as in the sometimes implicit sense of being coerced into collaborative mental health care by school actors or in expressions of network partners that (often unwantedly) resonate stereotypical or negative representations of parenting skills. These dynamics of power inequality may equally shape family’s motivation to engage with collaborative mental health care practice, as was illustrated in Ahmed’s case, where his father indicated to have agreed with care provision only because he felt it was impossible to voice disagreement within his son’s school, as a host societal institutional context. Our case analyses furthermore indicate how school partners and mental health partners sometimes work within different rhythms and time frames. School partners address the need for short-term solutions to be implemented within daily class practice whereas mental health partners address the therapeutic value of temporizing, through careful assessment, contextualization, validating the complex meanings of child development, parenting, and school trajectories within the family’s migration trajectory, and exploring dynamics within classroom and family–school interactions. As illustrated in Abdul’s case, school partners may for example contact the CCRC in moments of crisis or impending exclusion from school, hoping for an immediate solution and intervention. However, collaborative care at school requires a careful attunement between network partners and the CCRC in terms of pace when this seems necessary, and often this attunement requires continuous monitoring and negotiation.

This article forms a first systematic description of collaborative mental health care practice for refugee children. By providing a comprehensive description of school-based collaborative mental health care, including detailed accounts of intervention processes and working mechanisms through clinical case analysis, this article contributes significantly to the existing literature consistently indicating the need for a more in-depth understanding of collaborative mental health care for refugee children (e.g., Rousseau et al., 2012; Nadeau et al., 2017, 2018). While providing this novel and multilayered evidence for school-based collaborative mental health care, as well as sparking reflection on some of the interventions’ central premises, this article equally has some limitations that should be noted. First, the clinical case analysis of processes and working mechanisms was performed on three cases only. Future analyses of a larger body of case work could yield further insights to the findings presented here. Second, clinical case analysis was carried out by the first, second and last author who are part of the same clinical team and, as such, involved in each other’s cases through case collaboration and team supervision. While this co-development of analysis forms a strength of our study, it could be interesting to consider how interpretations and conclusions may alter if analysis also invites the views and voices of researchers or clinical professionals who are not a member of our clinical team. Equally, including the views and voices of school teams and refugee families might serve to broaden our analytical perspective. Third, descriptive exploration of school-based collaborative mental health care intervention is rooted in a developing practice in schools in the particular geographic region of Leuven, Belgium, and within the reality of a shared political support base between the city and university of Leuven and a particular urban integration policy that characterizes our collaboration with the municipality of Leuven in developing this practice of school-based collaborative mental health care. As such, this article’s findings cannot be generalized to other school-based collaborative mental health care practices as a matter of course.

In further stages of intervention development, other types of research could serve to continue advancing the intervention’s evidence-base (Veerman and van Yperen, 2007; van Yperen et al., 2017), including an exploration of these relational complexities in counteracting stigma and power disparities. First, a more systematic analysis of a larger body of clinical cases could grant further insight into collaborative mental health care’s central processes and working mechanisms. Second, qualitative interview or focus group research (e.g., with the child, the family, school actors or cultural brokers) and both qualitative and quantitative monitoring of collaborative care network meetings could contribute to an in-depth understanding of network partners’ lived experiences of the intervention, of intervention processes and outcomes. Third, the evidence on collaborative mental health care outcomes could be furthered by a multi-informant questionnaire study of children’s development, school functioning and mental health across time. Additionally, robust evidence on the effectiveness of collaborative mental health care could be generated through an experimental research study, whereby children receiving the intervention are compared to their peers in a control, a treatment-as-usual or alternative intervention condition. Last, further research could strengthen the proposed model for the joint assessment of child development within collaborative mental health care (see also: Figure 1; Based on: De Haene and Rousseau, 2020). As a seminal but growing body of studies points to interactions between refugee children’s mental health, linguistic competence, and social and cultural integration (e.g., Evans et al., 2020; Walker and Zuberi, 2020; Spaas et al., 2021), it seems highly relevant to further develop our understanding of refugee children’s psychosocial, language, and cultural development within the collaborative care context. Future research could, for example, engage in a profiling of prototypical associations between refugee children’s mental health, their linguistic development (both native language and second language proficiency) and patterns of social, home and host cultural integration, including a contextualization of development within the dimensions of family functioning, migration history, and host society conditions (Nadeau et al., 2018). Insight into these profiles of development could strengthen processes of assessment and intervention in collaborative mental health care intervention, and support schools in shaping teaching and care practices for refugee pupils. In general, the continued and scientifically supported development of our collaborative mental health care practice within the context of Leuven and a broadening of the practice to other geographic regions could serve to support expertise building in school systems and health care professionals, ultimately contributing to a national anchoring and the sustainability of this promising form of mental health care for refugee children and their families.

## Data Availability Statement

Questions regarding the datasets should be directed to CS, caroline.spaas@kuleuven.be.

## Author Contributions

CS participated in the design of this study, contributed casework, participated in analysis and drafted this manuscript together with LDH. SV provided and developed school-based collaborative mental health care in Leuven, Belgium, contributed casework to this manuscript, participated in analysis, and collaborated in the development of different drafts of the manuscript. SdS, RK, and LM reviewed and reflected on different drafts of the manuscript. LDH supervises and guides the ongoing development of school-based collaborative mental health care in Leuven, Belgium, designed the study, contributed casework, participated in analysis and drafted this manuscript together with CS. All authors read and approved the final manuscript.

## Conflict of Interest

The authors declare that the research was conducted in the absence of any commercial or financial relationships that could be construed as a potential conflict of interest.

## Publisher’s Note

All claims expressed in this article are solely those of the authors and do not necessarily represent those of their affiliated organizations, or those of the publisher, the editors and the reviewers. Any product that may be evaluated in this article, or claim that may be made by its manufacturer, is not guaranteed or endorsed by the publisher.

## Footnotes

1 The presented cases have been anonymized in order to ensure confidentiality for the families and other network partners.
